# Supplementary material for: “They are not HIV treatments drugs; they are preventive drugs (PrEP)”. Experiences of PrEP uptake among vulnerable adolescent girls and young women in Tanzania
Source: PLoS One. 2025 Jan 8;20(1):e0313501. doi: 10.1371/journal.pone.0313501 (PMC11709303; doi:10.1371/journal.pone.0313501)
Supplement: S1 File — (DOCX) [file pone.0313501.s001.docx]

**Supporting file 1: Consolidated criteria for reporting qualitative studies (COREQ): 32-item checklist**

| **Consolidated criteria for reporting qualitative studies (COREQ): 32-item checklist:** [COREQ (Consolidated Criteria for Reporting Qualitative Studies) -](https://onlinelibrary.wiley.com/doi/10.1002/9781118715598.ch21) [Guidelines for Reporting Health Research: A User's Manual - Wiley Online Library](https://onlinelibrary.wiley.com/doi/10.1002/9781118715598.ch21)  **Study Title: *“They are not HIV treatments drugs; they are preventive drugs (PrEP)”.* Experiences of PrEP uptake among vulnerable adolescent girls and young women in Tanzania** | | | |
| --- | --- | --- | --- |
| **No.** | **Item** | **Guide questions/description** | **Page number/paragraph and section of manuscript which reports the information that meets the criteria of the checklist** |
| **Domain 1: Research team and reflexivity** | | | |
| **Personal Characteristics** | | | |
| 1. | Interviewer/facilitator | *Which author/s conducted the interview or focus group?* | Page 5 line 177-179, Methods section (data collection sub-section) |
| 2. | Credentials | *What were the researcher's credentials? E.g. PhD, MD* | Page 5 line 177-179, Methods section (data collection sub-section) |
| 3. | Occupation | *What was their occupation at the time of the study?* | Page 5 line 177-179, Methods section (data collection sub-section) |
| 4. | Gender | *Was the researcher male or female?* | Page 5 line 177, Methods section (data collection sub-section) |
| 5. | Experience and training | *What experience or training did the researcher have?* | Page 5 line 177-188, Methods section (data collection sub-section) |
| **Relationship with participants** | | | |
| 6. | Relationship established | *Was a relationship established prior to study commencement?* | Page 4 line 157-164, Methods section (Sampling strategy sub-section) |
| 7. | Participant knowledge of the interviewer | *What did the participants know about the researcher? e.g. personal goals, reasons for*  *doing the research* | Page 4 line 157-164, Methods section (study design setting and population, sub- section) **Supporting file 4: Interview guide**  **Supporting file 5: Study informational sheet** |
| 8. | Interviewer characteristics | *What characteristics were reported about the interviewer/facilitator? e.g. Bias, assumptions, reasons, and interests in the*  *research topic* | Page 5 line 179-188, Methods section (Data collection section) |
| **Domain 2: study design** | | | |
| **Theoretical framework** | | | |
| 9. | Methodological  orientation and Theory | *What methodological orientation was stated to underpin the study? e.g. grounded theory, discourse analysis, ethnography, phenomenology, content analysis* | Page 4 line 133-136, Methods section (Study design sub-section) |

| **Participant selection** | | | |
| --- | --- | --- | --- |
| 10. | Sampling | *How were participants selected? e.g. purposive, convenience, consecutive,*  *snowball* | Page 4 lines 157-164, Methods section (Sampling strategy sub-section) |
| 11. | Method of approach | *How were participants approached? e.g. face-to-face, telephone, mail, email* | Page 4 line 157-164, Methods section (Sampling strategy sub-section) |
| 12. | Sample size | *How many participants were in the study?* | Page 5 lines 157-176, Methods section (Data collection sub-section) |
| 13. | Non-participation | *How many people refused to participate or dropped out? Reasons?* | N/A all participants who were invited to take part in the study after they were listen to the study information sheet, they all agreed to take part. |
| **Setting** | | | |
| 14. | Setting of data collection | *Where was the data collected? e.g. home, clinic, workplace* | Page 4 to 5 lines 166-169, Methods section (data collection sub-section) |
| 15. | Presence of non-  participants | *Was anyone else present besides the*  *participants and researchers?* | Page 5 lines 166-169, Methods section (data collection sub-section) |
| 16. | Description of sample | *What are the important characteristics of the sample? e.g. demographic data, date* | Page 6 to 7 lines 230-240, Results section |
| **Data collection** | | | |
| 17. | Interview guide | *Were questions, prompts, guides provided by the authors? Was it pilot tested?* | Page 5 lines 189-202, Methods section (data collection sub-section)  **Supporting file 4: Interview guide** |
| 18. | Repeat interviews | *Were repeat interviews carried out? If yes,*  *how many?* | N/A - No, it was not appropriate to conduct repeat interviews for this study  design. |
| 19. | Audio/visual recording | *Did the research use audio or visual recording to collect the data?* | Page 5, line 171-172, Methods section (data collection sub-section) |
| 20. | Field notes | *Were field notes made during and/or after the interview or focus group?* | N/A - No supplementary field notes were recorded other from the researcher's notes taken during interviews (notes of significant responses in case there was a failure in recording). Since this study does not follow an observational or ethnographic design, it is not necessary to include or reference field notes. If the items were indeed confiscated, we would provide a corresponding report. However, we believe it is not suitable to explicitly mention in the document that the items were not confiscated. |

| 21. | Duration | *What was the duration of the interviews or focus group?* | Page 5 line 170-172, Methods section (data collection sub-section) |
| --- | --- | --- | --- |
| 22. | Data saturation | *Was data saturation discussed?* | Page 6 line 193-202, Methods section (Data collection sub-section)  The initial predetermined sample size for in-depth interviews in each district was 10-20, as determined by budgetary planning. However, the saturation threshold was employed during the data collection process to determine when to cease. Collection of data within specific study domains. |
| 23. | Transcripts returned | *Were transcripts returned to participants for comment and/or correction?* | N/A - No, Due to the project's limited finance and small scale, we faced several constraints. There is insufficient funding available to reimburse participants and obtain their comments on their transcripts. However, we concluded that this study topic, design, and participant group did not warrant the use of participants' time and project resources. |
| **Domain 3: analysis and findings** | | | |
| **Data analysis** | | | |
| 24. | Number of data coders | *How many data coders coded the data?* | Page 5 lines 177-180, Methods section (Data analysis sub section) |
| 25. | Description of the coding tree | *Did authors provide a description of the coding tree?* | No – the overall coding framework reflected the research priorities of the  wider project, and this paper presents a subset of these results which cut across different sections and levels of the overall coding framework |
| 26. | Derivation of themes | *Were themes identified in advance or*  *derived from the data?* | Page 5 lines 212-220, Methods section (Data analysis sub section) |
| 27. | Software | *What software, if applicable, was used to manage the data?* | Page 5 line 205-206, Methods section (Data analysis sub section) |
| 28. | Participant checking | *Did participants provide feedback on the findings?* | No - Due to the project's limited finance and small scale, we faced several constraints. There is insufficient funding available to reimburse participants and obtain their comments on their transcripts. However, we concluded that this study topic, design, and participant group did not warrant the use of participants' time and project resources. |
| **Reporting** | | | |
| 29. | Quotations presented | *Were participant quotations presented to illustrate the themes / findings? Was each quotation identified? e.g. participant*  *number* | Quotations are presented to illustrate themes throughout the results section (pages 6-16) line number 188-508, identified by participant number, condition and nationality, Results section |
| 30. | Data and findings consistent | *Was there consistency between the data presented and the findings?* | The findings were derived from the data, and data is embedded in presentation of each theme (pages 6-16) line number 188-508, Results section |

| 31. | Clarity of major themes | *Were major themes clearly presented in the findings?* | Reporting of findings (pages 6-16) is structured by theme, barriers for PrEP uptake and facilitators for PrEP uptake: results section. The latter two themes were grouped using the social ecological model sub themes such as the individual/personal, interpersonal, institutional. Figure 1, in the manuscript has been attached in page 7 to displays the flow of the presentation of the results |
| --- | --- | --- | --- |
| 32. | Clarity of minor themes | *Is there a description of diverse cases or discussion of minor themes?* | Results section (pages 6-16) in line number 188-508 |
