# Supplementary material for: “They are not HIV treatments drugs; they are preventive drugs (PrEP)”. Experiences of PrEP uptake among vulnerable adolescent girls and young women in Tanzania
Source: PLoS One. 2025 Jan 8;20(1):e0313501. doi: 10.1371/journal.pone.0313501 (PMC11709303; doi:10.1371/journal.pone.0313501)
Supplement: S3 File — (DOCX) [file pone.0313501.s003.docx]

**Barriers for PrEP uptake in healthcare facility among AGYW**

**The prerequisites for initiating PrEP**

*“When they told me that I need to be tested first, I wanted to stop reaching decisions of using PrEP because I thought what would happen if I was tested and then I was found to have HIV infection. That is it, testing was giving me a lot of fear” (P1, Age 17, Dar es Salaam)*

*I was just afraid for the first time that how they will see me because I am still young. I was also afraid to test because I knew I might be affected, and people would think badly of me. But the health worker advised me and I understood him and took the test. (P8, 16 years-Dar es Salaam)*

*so I lose them because they talk...they think ooh I don't know this and that, or you will find yourself arguing with him "ooh I'm healthy" but on testing they refuse to test...they are afraid” (P43, 23 years-Tanga)*

**Disbelief effectiveness of PrEP**

*“They don't believe it can prevent HIV, but PrEP is used in small amounts; a few percent is not much. I think because they don't know, they don't believe that the preventive medicine (PrEP) can work and that it prevents HIV; that is, they do not believe it works” (P38, Age 24, Tanga)*

*“she feels because he doesn't know they don't believe... Hmm... That the preventive medicine can work that it prevents others say hey man these are not the HIV pills we are given Yeah... They are worried. Hahhahaha HIV pills we have been given. Yes, stay like that, one said, I don't believe if it is true, yes, yes, it is true, if it is the ARV drugs themselves. Because they don't know ARV drugs, that is, they didn't agree” (P38, 24 years-Tanga)*

*“aaah, when I grow up and have an education, it's the same, but I grew up with a condom and I couldn't leave it even though I grew up and got an education, because you know the first time you are told that they are liars, they are really useful, they really protect you, you grow up if you don't believe in them, that's why I started using them both ways, now after you go to test you are told good answers then you continue you start to believe little by little Faith is Coming” (P20, 24 years-Dar es Salaam).*

*“Despite that, I had education about PrEP, but I could not stop using condoms because the first time they tell you about PrEP, it is as if they (HCF) are liars, and you think they are not useful. Now, after you go to the test, you are told good answers, and as you continue, you start to believe little by little that faith is coming” (P19, Age 24, Dar es Salaam)*

*“I was curious to know more because I was told that these drugs are good so I decided to use them to help me. I was afraid because I thought I had used a condom but I am not sure of all the percentages that they will help me, I thought it would be good to use PrEP. I have fears but not like the ones I had before. I'm a little worried... I still don't believe if they can protect me one hundred percent... I'm scared but not like I had before... Because I've been told that these drugs prevent infection” (P3, 17 years, Dar es Salaam)*

*“Don't you know that we as vijans are in the street, so at first I was sceptical, even many vijans in the street were telling us that we are being lied to that PrEP is like ARV, I was really scared. So I became afraid but later I became more interested because I was given advice that these drugs are for protection, and when I looked at the environment I live in, it is a dangerous environment. So now that I was given that advice, it became more interesting to me, I saw that I could use it and I became in good health. So that is one of the things that has attracted me”(P6, 17 years-Dar es Salaam)*

*“At first I was worried but now I don't have any worries……..I felt the medicine would not be safe for health……….You know after hearing it for the first time I wanted to know more, many I asked said there is no such thing except that the medicines may be a stimulant of getting HIV infection” (P7, 17 years-Dar es Salaam)*

**Interference of refill hours with working hours**

*“because for a while I was stuck at work, because you know at work at other times, the boss does not let you leave just like that” (P45, Age 23, Tanga)*

*“It has happened before because here, where I work, there are times when it becomes difficult to go out, so I cannot go to get PrEP services, although there are times when I have permission from work and the drugs are not available” (P9, Age 17, Dar es Salaam)*

*Afternoon.... Why in the afternoon....... Because in the morning young people who have already finished school and have are living independent and own our own. For example, in the morning I have towork until five o'clock, after that I work on my own. So the time I come to take PrEP is the time I am resting. (P1, Age 17- Dar es Salaam)*

**Financial constraints**

*“I was unable to go for refills because I did not have transportation fare, and the health facility is far from here” (P19, 20 years, Dar es salaam)*

*“There is a friend of mine who failed to get service because of the shortage of bus fares....she lives in Mbezi and from Mbezi to this point, their stations there are small, not big stations, so he must come here. Therefore, you find that the availability of PrEP is very limited, so you have to come here to the big health centres. So he fails, from them to coming here the cost is three thousand or four thousand shillings” (P1, 17 years-Dar es Salaam)*

*“I don't spend any money to get medicine at the hospital Traveling from here to there is expensive. Those expenses are usually my own, sometimes if I fail, I ask for help. (2.)That is, I would like to find it in my neighbourhood. But I can also go pick up anywhere. I would like to tell them or advise them to help them get the travel expenses” (P2, 17 years-Dar es Salaam)*

*“I usually walk or take a tax. About forty minutes or an hour. If I use a taxi I usually spend half an hour sometimes I don't have a fare(2) I pay the fare to and from. Going depends on the transport that I have used. If it is a bus, I usually spend one thousand shillings to and from, if it is a bus, I usually spend two thousand shillings” (P3, 17 years, Dar es Salaam)*

*“I use the fare….. I don’t have three thousand shillings at other times…..Maybe when my friends help me pay for the fare once in a while” (P4, 17 years -Dar es Salaam)*

*“I usually walk or use a motorcycle. About forty minutes or an hour…..If I use Dalalada, I usually spend half an hour or forty-five minutes…..Sometimes I miss the fare, I pay the fare to and from. Going depends on the transport that I have used. If it is a bus, I usually spend one thousand shillings to and from, if it is a bus, I usually spend two thousand shillings” (P5, 18 years -Dar es Salaam)*

*“There is a financial challenge because there comes a time when you don't even have the fare but you want to get to the health centre but you don't have the fare at all. You think if I ask him, maybe someone will ask you what you are going to do, so you just have to lie until he gets it for you. So the financial challenge is one” (P6, 17 years-Dar es Salaam)*

*“The barrier is the lack of fare to get to the health centre to increase the dose. The fare from here to the health centre has often confused me. I find it very difficult…..I have often asked friends and relatives, but I have also built a system of keeping small coins for myself with the aim of using them as fare when I need to increase the dose. .Going five hundred and returning five hundred which is equal to one thousand shillings” (P7, 17 years-Dar es Salaam)*

**Adherence to the pills**

*“For the first time it is not easy to agree with that thing (PrEP), it is not easy to directly accept PrEP to use it....at the very beginning I was wondering, just asking myself the purpose of the medicine, how do you take the medicine every day” (P22, 20 years, Dar es Salaam)*

*“Yes. The first day I heard that you should drink it every day, now I was wondering if I would be able to, but later it turned out okay....The first day it affected me, I was afraid, because when I go to take it there are many of us, as well as I was afraid that if I use it every day, I can the other I was wrong” (P3, 17 years, Dar es Salaam)*

*“Yes. The first day I was told that you have to drink every day, that question is bothering me a little, now I was wondering if I could do it but later it was fine”(P5, 18 years -Dar es Salaam)*

*“Yes. There are challenges, for example the matter of taking medicine at the same time, how to get to the hospital regularly, these are the things that made me hesitate to start using this service at first” (P8, 16 years-Dar es Salaam)*

*“The thing that affected me is the time, the time to swallow is a problem, that is, if you swallow every day for two hours or one hour, this affected my decision somewhat” (P12, 20 years-Dar es Salaam)*

*The thing that affected me is the time, the time to swallow is a problem that is that of everyone day you eat two hours or one hour, this influenced my decision somewhat” (P12, 20 years-Dar es Salaam)*

**Misconceptions about PrEP pills**

*And if you look at the drugs, a person just seeing them knows that this person is using ARV drugs, so now it became a challenge.......I just had to leave as I am, without giving any information to anyone” (P6, 17 years-Dar es Salaam)*

*“It depends on the understanding of the person who will be understood and whether they agree with it or not, because some say that it is not something to prevent, but it is a person who has already experienced the effects and is using it (PrEP). Therefore, others are growing to know that these are drugs used by affected people and not preventive medicine.” (P42, Age 21, Tanga)*

*I don't have friends who use it, my friends themselves see that the drugs are for HIV, so I lose them because they talk...they see, ooh, I don't know this and that, or you will find yourself arguing with him "ooh, I'm healthy" but on testing, they refuse to test...they are afraid..... I was in a restaurant eating with my friends, they came to test me but my friends said "test again, we didn't believe", they said "we can't test him, we have tested him not long ago". When I was given those pills, they said "we will not walk with you" and I said okay. I told them "you have to use these pills" and they said they don't, so I broke up with them. (P43, 23 years, Tanga)*

**Labelling of PrEP users**

*“There was a time when my friends came to my house and took a picture of my PrEP can and started announcing that I am affected because I use those pills (PrEP)” (P45, Age 23, Tanga)*

*“My fellow youth in the street told me that I could not use the medicine (PrEP)—the pills. When they found out that I am taking PrEP pills, they told me that I also have AIDS "UKIMWI" (P47, 24 years-Tanga)*

*“There are many people here, so you find someone else is afraid, he is afraid, he sees someone who sees him, I go, he knows I am going to take anti-retroviral drugs.... Therefore, areas should be set aside. Anti-retroviral drugs should be given in different places from where anti-retroviral drugs are given” ( P52, 19 years- Tanga)*

*“Aaah, there is no meaning in freedom, we mingle with those people who take ARV drugs, so when you get there, you have to see a doctor to ask how you are progressing, but the community around you all know that we use ARVs” (P51, 24 years-Tanga)*

*“I was just afraid to say that maybe people will think bad of me if it's not bad too I'm telling them!.....they will see me like me maybe this guy is selling himself, that's why he's using condoms. I'm not free because when I come I'm tight and I have to hide myself because if people see me they will also know that I'm a victim of the HIV virus” (P52, 19 years-Tanga)*

*“These PrEP services are available at the same time and area, with those of people who use (HIV)anti-infection drugs, that's where the challenge arises, when you meet someone, from the same street, so they automatically assume that I am also taking HIV drugs of infection. That's where the trouble begins, they start spreading the information that I've met him somewhere he's taking HIV anti-retroviral drugs” (P22, 20 years, Dar es Salaam)*

*“If I were to be told to choose, I would choose the remote environment because the local environment is a challenge and people's understanding is different, a person who knows you just by seeing you knows that you are affected so you are taking ARV, so I would like to choose even Bagamoyo” (P6, 17 years-Dar es Salaam)*

**Institutional barriers**

**Inadequate privacy**

*“Another challenge that I see is that if someone like this comes to take medicine, someone knows that maybe you have come to take AIDS medicine.For example, when you enter here, people know very well that this person is going to take HIV treatment medicine (ARTs). eenh of the unit, when it is not ARTs there are other preventative medicines (PrEP)” (P44, Age 24, Tanga)*

*“The environment is bad because when we take PrEP in the same area where people with HIV are treated; that's what they do." So if someone passes by from a distance, they will say you are in the same group, so I don't like it. I'm not free. The interaction is good, but I don't like it. Staying with people with HIV, even though we're all humans. It might even happen that your person (referring to your man) saw you sitting there (CTCs); he will not care; he will perceive that you are already infected. Yes, they stopped giving you money for food. He ran away from you while you were there to take preventive medicine that protected him too.” (P43, Age 23, Tanga)*

*“Taking immunosuppressants has changed my life dramatically. First of all, a person's attitude because when you go to take medicine you meet other affected people because when you get there and you are given it they see you as one of the victims because it has challenged me but it also gives me more strength and courage. Actually, the environment is persuasive, mmm, there is confidentiality between the patient and the person who provides you with care, so the environment is persuasive. I seem to go to the CTC to take medicine. It doesn't bother me because I know I don't have that. infection and I go there to take medicine to protect myself so it doesn't bother me” (P46, 20 years, Tanga)*

*“the environment is bad because when we treat the same people with HIV, that's what they treat, so if someone passes by from afar, he will see someone in the same group, so I don't like it...I'm not free....separated from them, we should be given our share not like this if you are told that this is a men's ward, this is a women's ward, so you will understand that this is an AIDS drug and this is a preventive drug interaction he's nice, but the thing I don't like is staying with people with HIV, even though we're all human..something you'll feel you don't like..because they have to take their part and we take our part, he might even pass your man.mmmh. He saw you sitting there, heeee, he didn't care, he knew the one already, the one mmh. Yes, stop being given money to eat. He ran away from you, or else you have taken the preventive medicine that protects him” (P43, 23 years-Tanga)*

*“Hmmm, there was an obstacle one day. I went and met someone who knew me better.. So then he didn't have any secrets, so it was very difficult to get there to take the medicine, but I made contact and was brought to the hospital when I was outside. I made contact with the doctor I told him who is the service provider that I have arrived there but I have failed. I am here on the road for one or two reasons. He understood me mmh and it is only one day” (P51, 24 years-Tanga)*

*“Mmmmh I would suggest that first the service provider should be closer Eeh and his office where he dispenses medicine because there is another time you can go there, maybe you will be told he is not there, wait, and that becomes a challenge mmh” (P51, 24 years-Tanga)*

*“There, for this service it would be a bit of a secret, because when some go there who don't know, that is, they put in their memories that they are AIDS victims. They know we're going to take the dose, so it's good to have some concealment” (P9, 17 years - Dar es Salaam)*

*“I would like it to be a confidential area so that when you go to take medicine, everyone in the hospital does not have to see you” (P11, 22 years-Dar es Salaam).*

*“If this person sees me taking this, he will know if I am taking ARV because other people do not have that knowledge, if you tell him, he will tell you that it is not really ARV, so it becomes a bit of a challenge” (P20, 24 years—Dar es Salaam)*

*“so here is the time to enter secrecy again, it is a challenge that the environment should continue to be confidential!, because the things themselves are still confidential, secrecy now needs to continue to be confidential, that's why I suggested that there would be packages, a person comes, he is given his luggage, he puts it in the packages even if he keeps it this way, one cannot know anything, but sister, if you put the loan here, someone knows that you have an infection*

*..mmh..the obstacle is two times I went to the afu and when I arrived I met someone who I knew so I had to sit and wait until he came out and I went to”(P29, 22 years- Dar es Salaam)*

**PrEP drug stockout**

*“But we went there (HCF) three times, and each time we went, the medicines were finished. First, when we went, there were so many of us, so we found a long queue. When it was our turn, they told us the medicine was finished and we should return the following week. When that date arrived, we went and were told again to return on a certain date, but we did not go back” (P9, Age 17, Dar es salaam)*

*“Well, they (PrEP)are not always there” (P1, Age 17, Dar es salaam)*

*“(1)I always take ….. so I ride in the car….Half an hour….Yes…. I went and found that the medicines had run out, when I came I couldn't find them, I was told they had run out. ... If the clinic is shown, PrEP should be filled in many places in the city and in the villages so that all people can get it.(2).I didn't get the pills I wanted last time, the attendants told me that the pills have run out....I felt bad but I had no choice because the medicines were not there” (P4, 17 years -Dar es Salaam)*

*“Overall, PrEP has changed my life because first of all I have confidence in myself, I have confidence in myself and I am very grateful for this service. But this service has been brought to us but the government has not yet improved it, because the government is heavily dependent on ARV tablets, which is why they are widely available, but PrEP tablets are not available as ARVs. For example, since I have used them, I have not been able to find them in time (P9, 17 years-Dar es Salaam)*

*“But the second time, the medicine was not much, it was like they were sedating, but I got it” (P10, 17 years-Dar es Salaam)*

*“It has happened before because when I work here, there are times when it becomes difficult to go out, so I cannot go to get PrEP services, although there are times when even if I get it, the drugs are not available. There was also a day when I couldn't get to the health center because I didn't have anything in my pocket, I didn't have the fare, so I postponed the trip, I stayed, the next day like that until the week was over and I found a health worker to help me, he told me to wait and he would bring it to me, that's how I got help” (P9, 17 years-Dar es Salaam)*

*“You find that the other time is running out, if you go to find it there, you are told to come the next day, you go, you find it.I continue to swallow the other things, while I am waiting for that while the others are already following me...like......weeks are passing by. .while I wait I continue to use a condom! (P22, 20 years - Dar es Salaam)*

*because if the days are over, if you go, you are told right now that there are challenges like this and that, the availability of medicine is becoming difficult, we just ask that they do their best not to miss it. (P27, 22 years - Dar es Salaam)*

*The day I went was not the day of those services. I was told to come on a certain day and I actually went and was given care. I felt bad because I looked at the medicines I had had run out. I was left with one pill and as I was told that the medicines are important, so I must feel bad for missing them. But It didn't take many days, I only stayed one day (P50, 22 years - Tanga)*

*here in the past there was a period when they were lost so when they were lost we were informed that we should be patient but for these hours the service has been good. They said because the ones that were brought were about to expire or expire so they found themselves only distributing them a little until they went to order again eh you were late It took about two months to arrive. (P61, 24 years-Tanga)*

**Turned away by HCF**

*“The day I went was not the day of those services. I was told to come on a certain day, and I actually went and was given care. .I felt bad because I looked at the medicines, I had run out....I had only one pill left. Since I knew the importance medicines are important, I felt bad for missing taking them(PrEP)” ( P40, Age 22, Tanga)*

*“It was the first days when you went, they used to tell us that today is not the date to come on a certain day. I went and was told that today is not the date to take the medicine. Although I had already lost my the bus fair, I had to be satisfied now, what should I do, you can't fight, you can't fight with them, that's it, I can't sue them, it's me and my health to protect myself” (P61, 24 years-Tanga)*

**Long waiting times**

*"I see that the services are not good, it is challenging, because there are delays in getting the service. The lack of nurses causes us to take a long time, but if there were more nurses, the procedures would be done more quickly" (P3, Age 17, Dar es salaam)*

*“Maybe it's a queue. There are many of us, so the queue becomes big, so if you come late, you may find yourself in a queue that affects you until nine o'clock or eight o'clock and you are still here” (P13, 18 years - Dar es Salaam)*

*“It's just a queue. Another challenge is that there are few waiters, so they should be added so that when I get there, I am served faster” (P15, 21 years, Dar es Salaam)*

*“Nope. Maybe a queue. There are long queues and there are many of us even though we all need the same type of service. The challenge is queuing, many say they spend a lot of time, while they want a little time to spend on their duties” (P16, years- Dar es Salaam)*

*“no, because here you can come and meet a crowd of people, there are many people, so you can't get the service quickly mmmh mmhmmh so I think it will be difficult to get the service here” (P51, 17 years, Tanga).*

*“That is, what I was asking you to know is that if you go for the first time on the issue of PrEP, it is disappointing, that is, you may decide to just stop whatever it is. That is, when you arrive at the reception, it is not that you are sick, that is, the doctor just approves you to go to the CTC and take PrEP, but now it is a long chain, twisting and turning, that is, if you don't meet on Monday, that is, you will stop, you will sit there at the doctor waiting, that is, many people until it is disappointing. So now I was mainly asking the hospitals or clinics to check what the problem is with this person, if it is a PrEP issue, let him go in and get the medicine because that is a matter of hours. If he is late, he is at greater risk. Even if you go to CTC for medicine, when you get there, they will check you, that is, give you first priority” P6, 17 years-Dar es Salaam*

*“I feel like the service is not good, that is, it is challenging, because I am late to get the service....I should not stay too long. In other words, it should not exceed fifteen minutes....Addition of a nurse. The lack of nurses causes us to take longer, but if there were more nurses, the procedures would be done more quickly” (P3, 17 years, Dar es Salaam)*

*“That is, first of all, long queues, that is, doctors and nurses should be much faster to reduce the queues……. They(PrEP) should be released on time, that is, for the required time. For example, if there is a queue, you have to sit first and wait to be served, so they should improve” (P4, 17 years, Dar es Salaam)*

*(but we used it about three times, every time we went, the medicine had not run out.First, when we went, there were many of us, so we found a queue until we were reached. When that date arrived, we went and were told again to go on a certain date, but we didn't go, so we had to talk to the person who brought us the education to explain the challenges (2.) because there is always a queue on other days. So if the center is allocated for this service, the number of staff would increase to five or six, because there are many people who need the service” (P8, 16 years-Dar es Salaam)*

***Distance to the HCF***

“There are those who get services in nearby places and there are those who come from distant places. There are those who spend even two hours on the journey just to reach the health center” (P5, Age 19, Dar es Salaam).

*“When you look at the environment, for example, I can't be living here and go to the nearest healthcare facility to take PrEP, people will misunderstand me, you know?........but going to healthcare facility far away it's becoming normal here. Around hear (Place of residence) people will start to judge that I have started to eats HIV treatment drug (ART) and is not preventive medicine (PrEP)” (P20, Age 24, Dar es Salaam)*

*“That is, I would like to find it(PrEP) in my neighbourhood. But I can also I can go to pick up anywhere....The stations should not be far because I will have to use transport fee” (P2, Age 17, Dar es Salaam)*

*“I usually walk or use a stroller. About forty minutes or an hour…..If I use Dalalada, I usually spend half an hour or forty-five minutes…..Sometimes I miss the fare….........I normally, I pay the fare to and from. Going depends on the transport that I have used. If it is a bus, I usually spend one thousand shillings to and fro, if it is a bus, I usually spend two thousand shillings. (2)When I see that the day of going to the medicine arrives and I don't have a fare, I just try my best and start the trip to the health center early. ….. Increase the places to take medicine” (P5, 17 years -Dar es Salaam)*

*(I usually walk or take a taxi……..Half an hour………The whole hour………If I missed the fare and the day to go to take the dose has arrived, then I have no other choice, I have to walk” (P8, 16 years-Dar es Salaam)*

**Facilitators for PrEP uptake among AGYW in HCF**

**Experienced benefit of PrEP**

*“I just liked to use it because I love it and care a lot about my health. That is, I don't want to get HIV infection. They (HCP) told me to use those drugs because they are good. They told me that according to the environment I live in, those drugs are good because I can have sex with a person with the HIV virus and I will be safe” (P8, Age 16, Dar es Salaam)*

*“(1)Speaking of benefits, it has given me more freedom to know that there is something to prevent me from getting HIV infection. (2.)Ahaaaaa…..the opinion is that the PrEP service is very good because it has reduced new infections, especially among young people. Because young people are the most vulnerable because we want big things, we want good things and we don't look at who we are in relationships with. But also most of us girls do not have the ability to tell our lovers to check our health. But if we use these drugs, we get a chance to protect ourselves more and protect the person we will meet” (P1, 17 years-Dar es Salaam)*

*“Ahaa... the first thing that attracted me was that there is a possibility to reduce HIV infection, we young people are very vulnerable to HIV infection. If you think about it right now, there is no AIDS but there is the HIV virus, and you cannot tell a person who has the HIV virus by looking at them. If you consider we meet different people whose health conditions we do not know” (P1, 17 years-Dar es Salaam)*

*“I see the biggest advantage is not being infected with the HIV virus as well as getting rid of the fear of being infected is what made me use PrEP” (P4, 17 years -Dar es Salaam)*

*“That is, PrEP has really attracted me, because we as young people sometimes go through very difficult, dangerous situations. You know, as young people, you may be dating someone but the issue of testing is not there, you just look at his appearance and say that this man will not have a penis, at the end of the day you have sex with him but you don't know his health. I can say that these drugs are good because they give you the assurance of not getting an infection. The most interesting thing is that you can use these drugs even if your partner doesn't want to, because you can use them without him knowing, this is completely different from other methods like using condoms because you both have to agree” (P5, 18 years -Dar es Salaam)*

*“That is, that PrEP has really interested me, because we as young people sometimes go through a very difficult environment, a dangerous environment. You know, as young people, you can be dating someone but the issue of testing is not there, you just look at his appearance and say that this man will not have a penis, at the end of the day you have sex with him but you don't know his health” (P6, 17 years-Dar es Salaam)*

*“(1.)I can say that PrEP is very beneficial because it prevents infection so that you don't get affected. That alone is very important to me because it makes me free and able to have sex without the fear of infection, in short I can say PrEP is a good drug, if anyone is educated about these drugs, they must be interested in using them. (2.) My life is as usual, the only thing I can say that PrEP has changed is in terms of thinking and fear. At first I used to believe that the HIV virus is unstoppable, that is, if you just walk with someone who has the HIV virus, you must also be affected, but I no longer have that fear. I have changed my attitude and believe that if I am careful with the use of these drugs then I cannot be a victim at all..As I said before, PrEP is beneficial because it protects me from HIV infection. This is what made me decide to use PrEP”(P7, 17 years-Dar es Salaam)*

*“I just liked to use it because I love it and care a lot about my health. That is, don't let me get HIV infection....Yes. They told me to use those drugs because they are good…….They told me that according to the environment I live in, those drugs are good because I can have sex with a person with HIV and I will be safe…..As you know, we are young people, there are times when we have to have sex with different people so long as we can support ourselves or run our lives. This is the environment I mean. (2.) It is that it is free, that is, it is a free service. But it is also just to be told that I will be safe, I will not be able to get infected with HIV even if I date someone who is already a victim of HIV” (P8, 16 years-Dar es Salaam)*

*“What attracted me the most is because I liked to use it because it is a protection that protects me” (P9, 17 years-Dar es Salaam)*

*“For my safety, as you know health is everything. There are many challenges, that is, when I heard about those drugs, I was interested because of my safety. I really like to use those medicines, I always meet someone even if it's fake, they don't want to use a condom, but I know I already have immunity in my body”(P10, 17 years-Dar es Salaam)*

*“It is to protect oneself from AIDS, sex, other infections” (P11, 22 years -Dar es Salaam)*

**Adequate PrEP knowledge.**

*"Mmmh... actually what attracted me was that I was satisfied with the information the doctor gave me while giving me medicine (PrEP), so I saw that it was the right way for me" (P14, Age 19, Dar es Salaam)*

*I have no worries …..I have no fear ………Because I have been told that these medicines prevent infection. So I don't have the same fear of infection even if I meet someone with the HIV virus I will still be safe....I was afraid because I thought I had used a condom but I am not sure of all the percentages that they will help me, I thought it would be good to use PrEP(P5, 18 years -Dar es Salaam)*

*Yah, the presence of PrEP has changed my life, because before I was very worried, that is, I was saying that now that I am out with this person, I will be affected, but when I went to the hospital, I got good advice and I have used it to get tested and I became negative, that is, my life is still as good as my dreams are still progressing. P6, 17 years-Dar es Salaam*

*“Personally, I think it was the education I was given that convinced me to start using preventive medicine (PrEP)...eeeh education and advice from my friend” (P47, 24 years-Tanga)*

*Hmmm....actually what attracted me was that I was satisfied with the information the doctor gave me while giving me medicine, so I saw that it was the right way for me. (P14, 19 years-Dar es Salaam)*

*that is, the first thing I need to do is test and find out where I am... I found that I am fine, I have nothing, so I started using it... you are afraid, but that's how you grow, you are encouraged ahahah... I checked and I got good advice from the attendant that these medicines do not have any harm....... for me or for the future ...I used it....that is, because I listened well to the service provider....and I read it well about the things to take into account my assigned duties...so I read it and understood him...so I saw that there is no eeh for me (P25, years, Dar es Salaam)*

*“then I decided to use PrEP because of the information we were given, I saw that the information we were given about PrEP is what I thought it was better to just take and use” (P30, 22 years- Dar es Salaam)*

*The person who convinced me really guided me very well... He guided me and someone saw them and said they were HIV drugs... So I explained to him and he also said there is no problem and I want them. (P48, 21 years- Tanga)*

*What I advise is education, education should be given to those young people, they should be educated to know the importance of using preventive medicine, only education should be given more and more. Personally, I think it was the education I was given that convinced me to start using preventive medicine...eeeh education and advice from my friend. even contribute to the fare, because when his day comes he is not worried, he just goes to pick it up. (P37, 24 years- Tanga)*

**Having multiple partners**

*“I'm motivated to continue using PrEP because I'm myself, that is, I don't have one partner, Eeenh, that is, I don't really have a permanent” (P37, 24 years-Tanga)*

*“I just liked to use it because I love it and care a lot about my health. That is, don't let me get HIV infection. Yes. They told me to use those drugs because they are good. They told me that according to the environment I live in, those drugs are good because I can have sex with a person with HIV and I will be safe. As you know, we are young people, there are times when we have to have sex with different people so long as we can support ourselves or run our lives. This is the environment I mean” (P8, 16 years-Dar es Salaam)*

*“For me it's beneficial because I'm a walker, I'm a watcher and as you can see I'm a girl, I sleep with this and that, and the condoms themselves, as you know, are not very careful. For me it is beneficial” (P11, 22 years -Dar es Salaam)*

*“I noticed because I am a person who jumps around with different men. I saw that this could help me because it protects me from infection” (P12, 20 years-Dar es Salaam)*

*“Nope. My life is as usual... I mean, what I can say is that at the moment I am very confident that I have immunity in my body, I cannot get an infection. I no longer have the fear I had before. (2.) I was worried about my health……I felt that if I don't take medicine then I will be at risk of getting an infection because wherever I go around I meet various people whose health I don't know”(P12, 20 years-Dar es Salaam)*

*“The thing that attracted me to use preventive medicine was that I was motivated because it prevents me from getting HIV infection, I was motivated because I am not married, so I could be with this one tomorrow and this one, so in meeting those people, we scratch in different environments, so I was motivated like that” (P51, 24) years-Tanga)*

*“because I myself am not calm, I am calm, but someone might happen like this or you went to a bar and someone came up to you, a club and you had your vibes, you are drunk, you don't understand yourself, you just happen to do that act, you just can't use it” (P43, 23 years- Tanga )*

*“I mean, because I was there, I was walking with many men as a way to increase my income... he was there well, I feel good and I am growing at peace. I was here I walk with different men, that's why I come home one day and I don't come back the next day, so they went to my sister Ashura and directed her and she told me to come here on the third week and come pick it up” (P35, 16 years- Dar es Salaam)*

**Perceived HIV risk due to the nature of the work**

*“What led me to say let me use it is because of the work we do we encounter many challenges when we are at work, we meet people we do not know, and we cannot test them to know if they are sick or healthy. So, when I got the information about these medicines (PrEP), I was grateful because they protect me, then before using I checked my health and found that I was fine so I used it with confidence, and I know that I am using it to protect myself” (P9, Age 17, Dar es Salaam)*

*PrEP attracted me because I myself am in a dangerous environment. so I saw that you can meet someone else...he doesn't want a condom. .heh I said these pills then they will grow and it's good for us (P23, 24 years-Dar es Salaam)*

*it's that I was interested and I said let me go and get protection from diseases because of my job…..I don't have one man,…. That's why I said let me go and take it to protect myself from diseases... after I heard it, I wanted to know more first how it will help my body, and how it will protect me? Yes, I wanted to know if (P52, 19 years- Tanga)*

*Due to the work I do, the reason I was told because you find your man immediately you move and he doesn't even have a condom. So when I was told, you drink even if you don't have a condom, you use a condom. (P50, 23 years - Tanga)*

*What led me to say let me use it is because of the work we do we encounter many challenges when we are at work, we meet people we do not know and we cannot test them to know if they are sick or healthy. So when I got the information about these medicines I was grateful because they protect me, then before using I checked my health and found that I was fine so I used it with confidence, and I know that I am using it to protect myself. (P9-, 17 years - Dar es Salaam)*

*Ahaa... the first thing that attracted me was that there is a possibility to reduce HIV infection, we young people are very vulnerable to HIV infection. If you think about it right now, there is no AIDS but there is the HIV virus and you cannot tell a person who has the HIV virus by looking at them. If you consider we meet different people whose health conditions we do not know (P1, 17 years-Dar es Salaam).*

*Ha ha ha ha……. It's because of the fear of HIV infection…….The nurse told me it's good to know my health so that I can find out if I have an infection and I can use medicine so that I don't get to that stage which is worse.(P2, 17 years-Dar es Salaam)*

*I have no fear….Because I have been told that these drugs prevent infection. So I don't have the fear of being infected, even if I meet someone with the HIV virus, I will still be safe....I see the biggest advantage is not being infected with the HIV virus as well as getting rid of the fear of being infected.(P5, 18 years -Dar es Salaam)*

**PrEP ensured privacy**

*“That is, PrEP has really interested me, because we as young people sometimes go through a very difficult environment, a dangerous environment. You know, as young people, you may be dating someone, but the issue of testing is not there, you just look at his appearance and say that this man will not have a HIV infection, at the end of the day you have sex with him, but you don't know his health. I can say these medicines are good because they give you the assurance of not getting an infection. The most interesting thing is that you can use these drugs even if your partner doesn't want to, because you can use them without him knowing, this is completely different from other methods like using condoms because you both have to agree” (P6, Age 17, Dar es Salaam)*

*I saw that it is better to use medicine than to use other protections. No one knows what you are using but you (2)That is, PrEP has really attracted me, because we as young people sometimes go through very difficult, dangerous situations. You know, as young people, you may be dating someone but the issue of testing is not there, you just look at his appearance and say that this man will not have a penis, at the end of the day you have sex with him but you don't know his health. I can say that these drugs are good because they give you the assurance of not getting an infection. The most interesting thing is that you can use these drugs even if your partner doesn't want to, because you can use them without him knowing, this is completely different from other methods like using condoms because you both have to agree.( P5, 18 years -Dar es Salaam)*

*I really like to use those medicines, I always meet someone even if it's a joke, he doesn't want to use a condom, but I know that I already have immunity in my body. There were challenges, especially in thinking that maybe these drugs could be harmful, but as you know, there are tests to test for AIDS, but some do not want or are reluctant to use them, I thought and saw that this method of PrEP is more reliable. P10, 17 years - Dar es Salaam)*

*My safety..Just restraining myself...Because I'm still a girl, I can't know anyone...Others you can tell him let's go and check his health, he's boycotting you..you can't know someone's soul....Or sometimes you love him, you can't miss him....I'd better protect myself, ivo.(P49, 19 years- Tanga)*

*What attracted me because it is true that I am in a dangerous environment and also if you use those medicines you know that you are protecting yourself so that you do not get infected with the AIDS virus. And it's also because I don't have a single partner and I can't, maybe that person for a short time, I don't know if you understand someone, you found him right then and there. Right now, I can't measure him with me as my problem is not money, it's just fixed....Because I saw that you can use it to protect yourself and I found it very good.(P45, 23 years- Tanga)*

*I found it easy, it means you just drink it, not like the one where you persuade someone, this is what I drink myself. But the condom is until you convince your partner to agree to use it too. (P15, 21 years-Dar es Salaam)*

*Ahaaaaa…..the opinion is that the PrEP service is very good because it has reduced new infections, especially among young people. Because young people are the most at risk because we desire big things, we desire good things and we don't look at who we are in relationships with. But also most of us girls do not have the ability to tell our lovers to check our health. But if we use these drugs, we get a chance to protect ourselves more and protect the person we will meet. (P1, 17 years-Dar es Salaam)*

*.As you know, we are young people and sometimes we have to make love with different people so that we can make a living or run our lives. This is the environment I mean.(P8, 16 years- Dar es salaam)*

*“I found it easy, it means you just drink it, not like the one you convince someone, this is what I drink myself. But the condom is until you convince your partner to agree to use it too” (P15, Age 21-Dar es salaam)*

*But also most of us girls do not have the ability to tell our lovers lets check our health status. But if we use these drugs, we get a chance to protect ourselves more and protect the person we will meet” (P1, Age 17-Dar es salaam)*

**Support from social networks.**

*“I heard people were talking on WhatsApp groups only, they were to be talking and talking. So, to me I found someone, and I went and asked him to explain to me about this because she is an adult and I told him then it's fine and I started using PrEP” (P32, Age 17, Dar es Salaam)*

*“but when I involved my friend, she encouraged me, I came back the next day and tested, and it was negative. That is when I found the strength to use PrEP”*

*(P1, 17 years-Dar es Salaam)*

*“My sister advised me that these medicines are good and do not have any side effects other than to protect us from viral infection” (P2, 17 years-Dar es Salaam)*

*“They should come to the health center to get more help. The community and relatives would also help the party to convince him and explain the importance of using PrEP as well as the users would be given various help including fares to be able to reach the health center” (P7, 17 years-Dar es Salaam)*

*“I asked for advice, I asked my friend for advice. …..I asked him for advice and he told me that it is better to use it, he told me that it is better to use it, prevention is better than cure, he told me that you should use it in case you vomit as long as the medicine you are using works if you see that you are overwhelmed, go back to the hospital. He told me even if you use panado if it is for vomiting you will just vomit” (P6, 17 years-Dar es Salaam)*

*“but when I shared with my friend about my decision to use PrEP, she encouraged me, I went back the next day and tested, and it was negative. That's when I found the strength to use PrEP” (P, 17 yeard – Dar es Salaam)*

*“hmm hmm... what should I do... because I, before my sister took me, I heard the advertisements, There is a preventive medicine, PrEP!.. there are pills that prevent infection, and if you look at our work, I said let me get involved and I started using PrEP” (P36, 24 years – Dar es Salaam)*

*“other of my friend said ok so I want to see you use it like in a month or a week and I will use it. There is a sister and we called her and we all started with my friend. He moved us again, he came to us” (P50, 23 years-Tanga)*

**Free availability of PrEP**

*“First, I saw that we are given for free, as well as that we are kept in a good environment of not being infected with the HIV virus whenever we have sex with an infected person” (P3, Age 17, Dar es Salaam)*

*“I think it is easy and convenient to use PrEP probably because I am close to where I go to take my medicine” (P46, Age 20, Tanga)*

*“It is that it is free, that is, it is a free service. But it is also just to be told that I will be safe, I will not be able to get infected with HIV even if I date someone who is already a victim of HIV” (P8, 16 years-Dar es Salaam)*

*“Don't be afraid if they don't have money to buy the condom, go and get the medicine (PrEP) for free” (P8, 16 years-Dar es Salaam)*

**Receiving refills reminders**

*“Something that makes it easier is sometimes they remind us of when the date is approaching, they call us. They send us a message as soon as the dates are approaching” (P15, Age 21, Dar es Salaam)*

*“There were still about three days left when I got a call, and he told me that the medicine was about to run out. If you're going to be a while, come and pick it up, don't wait until that date arrives, come pick it up, we'll be there for you” (P46, Age 20, Tanga)*

*They always send messages. They send the day before that day (P1, Age 17, Dar es Salaam*

*They call me or write in a card for me. They write me a date, that certain dates medicine will be over. so, I have to go pick them up. P2, Age 17, Dar es Salaam*

*They always send me messages. The same day that I am supposed to go and take the medicine. (P3, Age 17, Dar es Salaam)*

*Something that makes it easier sometimes they remind us of when the date is approaching they call us. They send us messages as soon as the dates are approaching (P15, Age 21-Dar es Salaam)*

*Something that makes it easier sometimes they remind us of when the date is approaching they call us. They remind me before the date arrives, they always use the mobile phone to contact me. (P16, 20 years, Dar es Salaam)*

*It had remained at least about three days. I got a phone call, and they told me that the medicines are about to run out. If you have time, come and pick it up or if you don't come and forget the payment, when the date comes, come pick it up and we will be there for you. (P46, 20 years- Tanga).*
