# Supplementary material for: “They are not HIV treatments drugs; they are preventive drugs (PrEP)”. Experiences of PrEP uptake among vulnerable adolescent girls and young women in Tanzania
Source: PLoS One. 2025 Jan 8;20(1):e0313501. doi: 10.1371/journal.pone.0313501 (PMC11709303; doi:10.1371/journal.pone.0313501)
Supplement: S4 File — (DOCX) [file pone.0313501.s004.docx]

**Supporting file 4-Interview guide for vulnerable adolescent girls and young women experiences of PrEP uptake in healthcare facilities in Tanzania.**

My name is ______________________________. Thank you for agreeing to take some time to talk to us today about oral PrEP for HIV prevention . Through this discussion, we would like to discuss certain, more specific topics on oral PrEP that we hope will help in understanding and improving the overall access and delivery of oral PrEP in Tanzania healthcare facilities. I am looking forward to hearing your thoughts on the questions I will ask you. Please know there are no right or wrong answers or opinions about the topics we are discussing, so feel free to share your thoughts openly. We’re simply looking to collect opinions, experiences, and beliefs from a range of men and women who participated in the earlier phase of this study. Also, feel free to skip any questions that you do not want to answer.

| **Section A: Biography**  **First, I will ask you general questions about yourself** | |
| --- | --- |
| 1. Please tell me a brief history of yourself to help me get to know you better. | **Guiding probes:**   1. where they grew up? 2. what they do for a living? 3. if they have family? 4. are they new to the area? 5. any other details that would help us to know you better.   **[Note: no need to ask all probes, keep this section fairly short]** |
| **Section B: PrEP Acceptability**  **I would like to know your knowledge of PrEP and acceptability** | |
| 1. I would like to confirm with you if you have used or still using PrEP? | 1. How long have you been using PrEP? 2. How did you hear about PrEP at first? 3. What do you know about PrEP? 4. What have you heard about PrEP 5. Who can take PrEP and why? 6. How is it taken? |
| 1. What interested you about PrEP? | 1. What made you want to learn more about PrEP? 2. What are other HIV prevention methods have you used in the past? Do you still use them (yes/ no) why? 3. Do you have any concerns about PrEP as a prevention method? 4. How has the introduction of PrEP changed your life? |
| 1. Acceptability of PrEP among vAGYW | 1. How did you decide if taking PrEP was right for you at the beginning, apart from other HIV preventive methods? 2. What were the pros and cons you considered when making the decision? 3. How did people in your life influence your decision about whether or not to access PrEP? (e.g. partner, family, or friends) 4. What concerns did you consider when making your decision? cost,acceptability 5. How did the requirements for getting PrEP and taking it regularly influence your decision of whether or not to access it?    1. ***[If this question is not clear, offer examples:]*** Accessing PrEP, time spent at the clinic; privacy; ability/willingness to take daily; forgetting; pill storage, pill burden, HIV testing |
| **Section C: Experience of Accessing PrEP**  Here, I would like for you to walk me through your experiences of accessing PrEP in the selected health care facility. | |
| 1. **Physical Accessibility;** I would like to know your experiences of getting to the health care facilities for PrEP related services. | 1. How do you get to the health care facilities to access PrEP related Services? 2. How long does it take to get to the health care facilities to get PrEP? 3. Did you take time off work? 4. Did someone come with you? 5. Are there any physical barriers/facilitator that that you have encounter that have made it easier or hard to get to get/attend to the health care facilities to access PrEP? |
| 1. **Availability:** Can you walk me through your experiences of accessing available PrEP services in the selected health care centre. | 1. Can you describe what happens when you go to the health centre/clinics for PrEP? 2. Can you tell me who is involved in providing PrEP?    1. What did the the health provider tell you?    2. Did you talk about any of your concerns about PrEP?    3. The interaction you had with the staff, did motivated you to continue to access PrEP in that particular health centre? 3. What part of the health centre/clinics do you go to get the services? 4. How easy or difficult was it to get services relating to PrEP once in the centre? 5. What made it easy or difficult? 6. How long did it take after you have arrived in the hospital and to be attended by the staff? 7. Did you get the services that took you there? Was PrEP available? If No, what did the health care provide told? 8. How did that made you feel? Where you satisfied with their answers? |
| 1. **Affordability:** Here I would like to understand more if there are ant financial cost associated with accessing PrEP. | 1. Do you incur any cost to obtain PrEP or refill? 2. What is the cost for? 3. Do you getr any finjancial support from the government/NGO,s or a person to help you access PrEP?Name of the person or organization providing support, how much do they give? 4. Is the financial support usually enough or you have to look. For alternative sources? 5. Can you work me through any incidences that happened to you or your friends were you failed to access PrEP due to financial contraist? 6. How did go about to solve the incidence? |
| 1. When accessing PREP in the selected government health facilities, what were the biggest challenges for accessing PrEP services? | [*If no challenges mentioned, skip to probe D*]   1. Did you ever miss a dose a refill visits because of these challenges? 2. What did you do in order to manage these challenges or seek help for them?( Probe :clinic, other sources) 3. What have you heard are other people(friends/collegues) is biggest challenges for accessing PrEP? |
| 1. What are the thing that made it easier for you to access PrEP in the.selcetd. Government Hospital? | 1. Do you have any ideas or suggestions about how access to PrEP for vAGYW could be made easier? |
| 1. If you have gotten refills, can you tell me your exiprences how was it when you went to the health care centre to get refills? | 1. Can you work me through your experience in making arrangements to go to the health centre for refill PrEP? 2. How long does it take between arriving at the health centre and getting your refills or PrEP? 3. Would you like to be able to choose a clinic location, time, nurses, or date of appointment? 4. What did the clinic, HCP peer navigators do to help you remember to attend your refill of PrEP? 5. What things have been helpful for you during your refills visit in the health care centre/ Clinics? 6. What things have been difficult for you during your refills visits? |
| **Section D: PrEP delivery in the future and changes to health care delivery.**  **In this session, I will like to discuss with you how PrEP should be delivered in the future to ensure that a larger number of vAGYW have easy access.** | |
| 1. What locations/places or environments will it be easier and safer to go and get PrEP? | - 1. How far are you willing to travel to get PrEP?   2. What hours will be easier to get PrEP for you?   - How far would you be willing to travel to get PrEP?   - How long would a reasonable waiting time be for a PrEP clinic appointment? |
| 1. How can the clinic experience be made better? | 1. How should the clinic procedures be made better? 2. How should the clinic space or environment be made better? 3. How should the procedures for refills be made better? |
| 1. What kind of information or counselling do you think could be helpful for vAGYW who would like to access PrEP? | |
| 1. What kind of social or financial support from partners, friends, family, young women need to help them access PrEP? | |
| 1. Have you stopped or thought of stopping using PrEP in the future? If so, what will be your reasons for this be? | |
| **Recommendation and suggestions** | |
| 1. Is there anything else that you would like to talk about relating to vAGYW experiences of accessing PrEP, that I have not covered? 2. Do you have anything you would like to tell me or ask me ? | |
