# Supplementary material for: “They are not HIV treatments drugs; they are preventive drugs (PrEP)”. Experiences of PrEP uptake among vulnerable adolescent girls and young women in Tanzania
Source: PLoS One. 2025 Jan 8;20(1):e0313501. doi: 10.1371/journal.pone.0313501 (PMC11709303; doi:10.1371/journal.pone.0313501)
Supplement: Attachment — Submitted filename: Review HIV PrEP Young people.docx [file pone.0313501.s005.docx]

**“They are not HIV treatments drugs; they are preventive drugs (PrEP) ”. Experiences of using PrEP among vulnerable adolescent girls and young women in Tanzania**

44 The introduction

45 of pre-exposure prophylaxis (PrEP) brought hope for changing the HIV cascade in the

93 Previous SSA studies

94 identified factors such as increased HIV risk perception, desire for HIV-negative status,

95 negative condom attitudes, peer influence, social support, education, awareness of PrEP

96 effectiveness and safety, and convenient service access free of charge (2,11–20).

97 barriers to PrEP uptake, even for highly interested individuals, include limited knowledge

104 without parents/guardians in diverse SSA contexts (30).

167 biases?

For the Results section I recommend to include a table with Participants’ characteristics? It can include Age, Sex, School level, PrEP status, etc.

212 Most participants in this study were 20–24, resided in Dar es Salaam, and were out of

213 school with a secondary education level. Around two-thirds had never had children, most

214 used condoms, and PrEP for HIV prevention and only few used PrEP only. The most

215 common occupation was petty business, food vending and bartending, and only a few were

216 unemployed. Participants reported that they access PrEP care in HCFs, and few reported

217 accessing care in dispensaries and hospitals.

304 limits them to accessing and initiating PrEP freely. The identified misconception also made

305 some worried and scared to initiate PrEP use at the beginning because people in their

306 social environment associated PrEP use with HIV;

460 particularly in situations where their partner declined to use a condom. Furthermore, a

461 significant number of participants in the study recognized that their motivation for

462 engaging in sexual relationships with several partners was primarily driven by the financial

463 support they received from these partners.

294 Interpersonal barriers

507 Interpersonal facilitators

519 The institutional facilitators reported by participants for continuing to access PrEP

520 included the factors of PrEP accessibility and receiving refills reminders, as elaborated

599 At the institutional level, similar to past research, the study has identified several problems

600 that hinder the uptake of PrEP.

600 These include instances of PrEP medicine stock-outs,

601 instances when HCP sends patients away, and extended waiting periods for care at HCF due

602 to a disparity between the number of staff and PrEP users (17,19,20) .

602 The latter relevance

603 can be attributed to the fact that in settings with limited resources, the problem of

604 accessing resources like drugs and staff in HCF is a common obstacle to seeking medical

605 attention
